# Supplementary material for: Novel Driver Strength Index highlights important cancer genes in TCGA PanCanAtlas patients
Source: PeerJ. 2022 Aug 11;10:e13860. doi: 10.7717/peerj.13860 (PMC9375969; doi:10.7717/peerj.13860)
Supplement: Supplemental Information 2 [file peerj-10-13860-s002.zip › COHORTS/genes plots/detailed/2021_8_16_14_9_distribution_events_detailed_males_1.pdf]

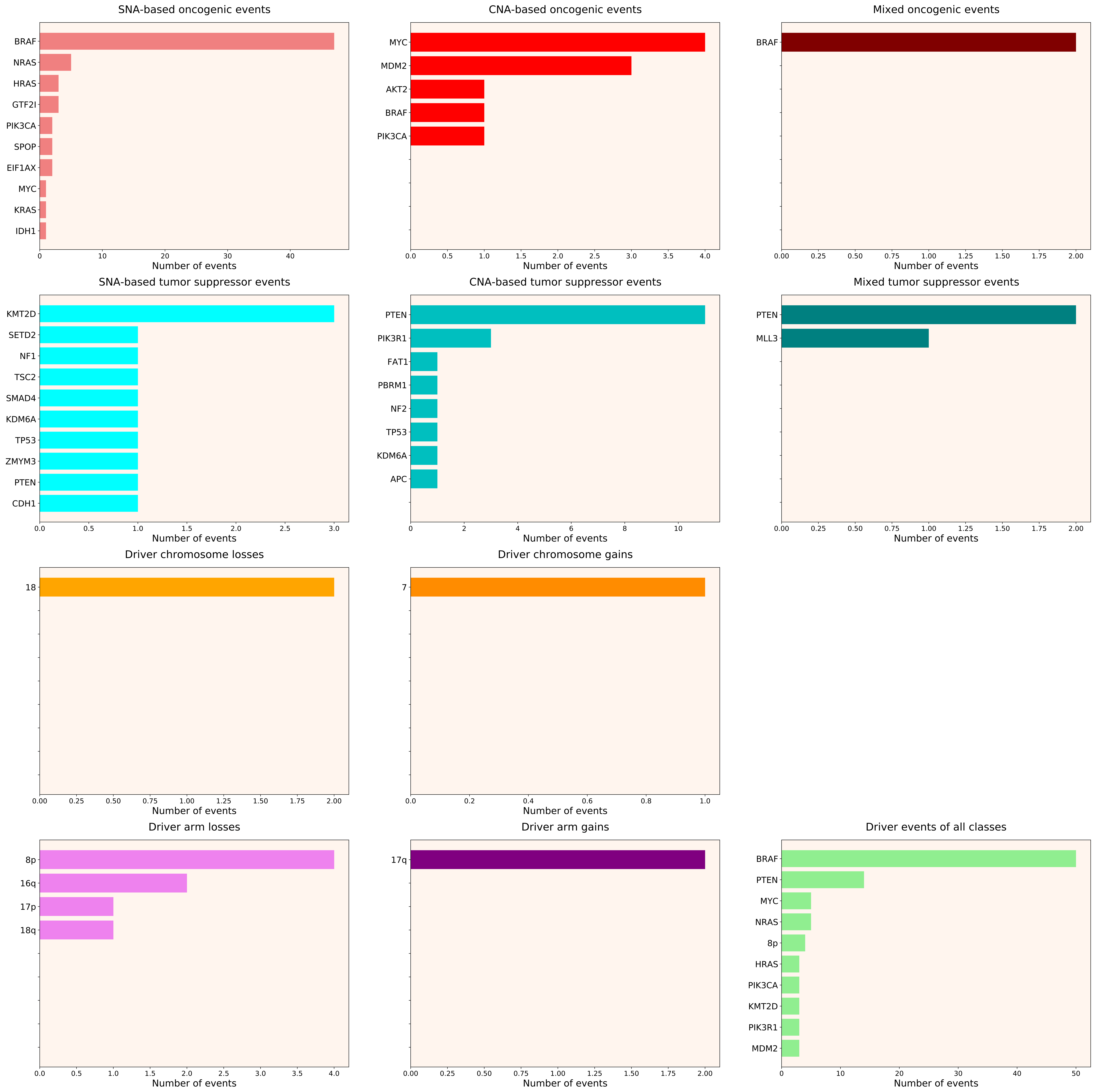
SNA-based oncogenic events
BRAF
NRAS
HRAS
GTF2I
PIK3CA
SPOP
EIF1AX
MYC
KRAS
IDH1
0 10 20 30 40
Number of events
CNA-based oncogenic events
MYC
MDM2
AKT2
BRAF
PIK3CA
0.0 0.5 1.0 1.5 2.0 2.5 3.0 3.5 4.0
Number of events
Mixed oncogenic events
BRAF
0.00 0.25 0.50 0.75 1.00 1.25 1.50 1.75 2.00
Number of events
SNA-based tumor suppressor events
KMT2D
SETD2
NF1
TSC2
SMAD4
KDM6A
TP53
ZMYM3
PTEN
CDH1
0.0 0.5 1.0 1.5 2.0 2.5 3.0
Number of events
CNA-based tumor suppressor events
PTEN
PIK3R1
FAT1
PBRM1
NF2
TP53
KDM6A
APC
0 2 4 6 8 10
Number of events
Mixed tumor suppressor events
PTEN
MLL3
0.00 0.25 0.50 0.75 1.00 1.25 1.50 1.75 2.00
Number of events
Driver chromosome losses
18
0.00 0.25 0.50 0.75 1.00 1.25 1.50 1.75 2.00
Number of events
Driver chromosome gains
7
0.0 0.2 0.4 0.6 0.8 1.0
Number of events
Driver arm losses
8p
16q
17p
18q
0.0 0.5 1.0 1.5 2.0 2.5 3.0 3.5 4.0
Number of events
Driver arm gains
17q
0.00 0.25 0.50 0.75 1.00 1.25 1.50 1.75 2.00
Number of events
Driver events of all classes
BRAF
PTEN
MYC
NRAS
8p
HRAS
PIK3CA
KMT2D
PIK3R1
MDM2
0 10 20 30 40 50
Number of events
